# Antibiotic Resistance Determinants in a *Pseudomonas putida* Strain Isolated from a Hospital

**DOI:** 10.1371/journal.pone.0081604

**Published:** 2014-01-17

**Authors:** Lázaro Molina, Zulema Udaondo, Estrella Duque, Matilde Fernández, Carlos Molina-Santiago, Amalia Roca, Mario Porcel, Jesús de la Torre, Ana Segura, Patrick Plesiat, Katy Jeannot, Juan-Luis Ramos

**Affiliations:** 1 Laboratorio de Investigación y Control Agroalimentario, Universidad de Huelva, Huelva, Spain; 2 Estación Experimental del Zaidín, Consejo Superior de Investigaciones Científicas, Granada, Spain; 3 Centro de Investigación en Química Sostenible, Universidad de Huelva, Huelva, Spain; 4 Bio-Iliberis Research and Development, Peligros-Granada, Spain; 5 Centre Hospitalier Régional Universitaire - Hôpital Jean Minjoz, Besançon, France; Imperial College London, United Kingdom

## Abstract

Environmental microbes harbor an enormous pool of antibiotic and biocide resistance genes that can impact the resistance profiles of animal and human pathogens via horizontal gene transfer. *Pseudomonas putida* strains are ubiquitous in soil and water but have been seldom isolated from humans. We have established a collection of *P. putida* strains isolated from in-patients in different hospitals in France. One of the isolated strains (HB3267) kills insects and is resistant to the majority of the antibiotics used in laboratories and hospitals, including aminoglycosides, ß-lactams, cationic peptides, chromoprotein enediyne antibiotics, dihydrofolate reductase inhibitors, fluoroquinolones and quinolones, glycopeptide antibiotics, macrolides, polyketides and sulfonamides. Similar to other *P. putida* clinical isolates the strain was sensitive to amikacin. To shed light on the broad pattern of antibiotic resistance, which is rarely found in clinical isolates of this species, the genome of this strain was sequenced and analysed. The study revealed that the determinants of multiple resistance are both chromosomally-borne as well as located on the pPC9 plasmid. Further analysis indicated that pPC9 has recruited antibiotic and biocide resistance genes from environmental microorganisms as well as from opportunistic and true human pathogens. The pPC9 plasmid is not self-transmissible, but can be mobilized by other bacterial plasmids making it capable of spreading antibiotic resistant determinants to new hosts.

## Introduction

Human disease outbreaks are increasing at an alarming rate. One of the most recent and serious occurred in Germany, involving a Stx2a-producing *Escherichia coli* (STEC) strain of serotype O104:H4 that caused more than 4000 cases of illness and 50 deaths. This strain exhibited resistance to numerous antibiotics making it difficult to eradicate [1]. Horizontal gene transfer has been proposed as the most likely genetic event for the spread of multidrug resistant phenotypes in pathogens [2]; however, a question that still needs to be answered is ‘What is the origin of these acquired antibiotic resistant determinants?’

*Pseudomonas putida* strains are typically found in soil and water and members of this species have a broad metabolic versatility, which allows them to adapt to different habitats and nutritional environments [3], [4], [5], [6]. Strains of this species have occasionally been isolated from patients in hospitals in Japan, the USA, Italy and France. Infections by these microorganisms have been reported to be linked to insertion of catheters or drainage tubes [7], [8]. Hospital isolates of *P. putida* are often resistant to β-lactams [9], [10], [11], and instance Yomoda *et al.* [11] reported that of 32 *P. putida* strains isolated in hospitals in Japan twenty two of them harbored plasmids transferable to *P. aeruginosa* by conjugation or transformation. The same study also indicated that a number of plasmids from these clinical isolates were responsible for resistance to aminoglycosides. Apart from the fact that opportunistic microbes could become ‘specialized’ pathogens able to attack the most vulnerable immunocompromised patients [11], [12]; the ability to transfer antibiotic resistant determinants from non-pathogenic species to pathogens in hospital environments is a serious concern [11], [12].

The Hospital of Besançon in France has established a collection of *P. putida* isolates from in-patients, and in agreement with von Greenitz and Weinstein [7], it has been found that these strains have a low pathogenic potential when compared with *P. aeruginosa* PAO1 using virulence assays in a insect model (our unpublished results). We analyzed 15 of these isolates and found that one of them, *P. putida* HB3267 (Hospital of Bensançon 3267), was able to kill insects and exhibited resistant to a large number of antibiotics. To shed light on the unusual pattern of antibiotic resistance of the strain HB3267 we sequenced and analysed the genome. The analysis revealed that a number of genes involved in multi-drug resistant phenotypes are located in a non-self-transmissible plasmid that was shown to be an efficient vehicle for spreading antibiotic resistance between different *Pseudomonas* strains.

## Materials and Methods

### DNA analysis and identification of the HB3267 strain

Amplification of 16S rDNA using HB3267 chromosomal DNA was performed with the F8 and R798 primers and the complete sequence of the gene compared with 16S rDNA sequences in databases [13]. Aranda-Olmedo *et al.* [14] showed that *P. putida* strains are characterized by the presence of a highly conserved 35-mer REP sequence. A primer based on the KT2440 REP sequence was used to amplify HB3267 DNA. Positive (*P. putida* KT2442, [15]) and negative (*Escherichia coli*) DNA controls were included. The REPc method allows identification of *P. putida* strains [14] since this primer amplifies only DNA from this species, producing products of different sizes for each strain. For multilocus sequence typing (MLST) we used a set of primers to amplify RNA polymerase sigma factor *rpoD*, DNA gyrase subunit B *gyrB*, N-(5′-phosphoribosyl) anthranilate isomerase *trpF, 6*-phosphogluconate dehydratase *edd*, and recombinase A *recA* genes [16], [17]. The complete gene sequences were obtained, translated into the protein sequence and compared as described [17].

### Antibiograms

For these assays 31 different antibiotics were used (Biomerieux commercial disk). Overnight cultures of HB3267 were spread on 240×240 mm LB plates, air dried in a laminar flow and then discs containing antibiotics were placed on the LB plates. Plates were incubated at 30°C for 16 h. Halos surrounding the discs were measured as an indication of inhibition of growth. This assay was repeated at least three times in duplicate.

### Minimal inhibitory concentration (MIC) assay

These assays were performed in 96-well plates, using LB medium and the following stock antibiotic solutions: tetracycline (Tc), 10 mg/ml; kanamycin (Km), 25 mg/ml; gentamicin (Gm), 100 mg/ml; nalidixic acid (Nal), 10 mg/ml; spectinomycin (Sp), 100 mg/ml; rifampicin (Rif), 10 mg/ml; chloramphenicol (Cm), 30 mg/ml, ampicillin (Ap), 100 mg/ml, norfloxacin (Nor), 20 mg/ml and ceftriaxone (Cro), 25 ml. Serial 10-fold dilutions of the stock antibiotic solutions were prepared and 10 µl of each of these dilutions added to 190 µl of LB, minimal medium M9 and Mueller-Hinton broth medium. Optically standardized 18 hour cultures (10 µl) of *P. putida* strains were used as inoculum. The 96-well plates were incubated at 30°C and 200 rpm overnight and culture turbidity was measured as an indication of growth. The MIC value was established as the lowest concentration at which an antibiotic inhibits growth >90% [18]. The assays were repeated three times in duplicate.

### Biofilm susceptibility testing

Biofilm assays were performed in 96-well flat-bottomed polystyrene microtitre plates. An aliquot of 100 µL of a bacterial suspension contains 10^5^ CFU/ml was added to each well and incubated for 5–6 h at 30°C. Subsequently, liquid culture medium was removed; the wells of the plates washed twice to eliminate all planktonic cells and finally serially diluted antibiotics in LB medium added. These plates were incubated overnight at 30°C, and after removing planktonic cells as above 100 µL LB was added, and the biofilm cells released by 5 minutes low intensity sonication (Branson 1510 waterbath ultrasonicator). The minimal biofilm eradication concentration (MBEC) was defined as the minimal concentration of antibiotic required to eradicate the biofilm [19].

### Conjugation experiments

*Pseudomonas putida* KT2440 (Tel), a tellurite resistant strain and *P. putida* HB3267 were grown overnight on LB medium.

For biparental matings 1 ml cultures with a turbidity of around 1 at 660 nm were mixed, harvested by centrifugation, wasted with LB and resuspended in 50 µl LB that was laid on nitrocellulose filter disks placed on LB plates. After overnight incubation, transconjugants were selected on LB medium containing Tel, Sm and Tc. For triparental mating experiments, receptor and donor strains were used as previously described, but the pWW0 [20] and pRK600 plasmids were used as helper plasmids.

### Sequencing

Genomic DNA containing both the chromosome and pPC9 plasmid was purified from strain *P. putida* HB3267, using the Wizard® Genomic DNA Purification Kit and sequenced using *454* technology by Macrogen (Seoul, Korea), and assembled into 278 contigs, providing 25× coverage. These contigs were ordered by comparison (BLASTN) with the genomic sequences from other *P. putida* available in the database (KT2440, NC_002947.3; F1, CP000712.1; GB-1, CP000926.1; W619, CP000949.1; BIRD1, CP002290.1), as well as with a close relative *Pseudomonas entomophila* L48 (NC_008027.1). Genomic gaps were closed by designing primers at the contig ends, followed by PCR and further sequencing of the junction sequences. Genomic DNA was automatically annotated using a program pipeline based on Glimmer 3.0 [21] for gene prediction, and BLAST and RPSBLAST for functional assignment of ORFs, based on sequence similarity to sequences deposited in the NR, Swissprot, COG, Pfam, Smart and Prk databases [22] Finally, automatic annotations were manually curated. The chromosome and plasmid sequences are available through Genbank under accession numbers CP003738 and CP003739, respectively.

## Results and Discussion

### Identification of strain HB3267

A collection of *Pseudomonas putida* strains isolated from humans has been established in the Bacteriology Laboratory of the University Hospital of Besançon (France). Among these, only one strain named HB3267 which was isolated from an in-patient who died from unknown causes, was able to kill insects (Porcel, M. and Duque, E., In preparation). Since *P. putida* strains are seldom isolated from humans a series of molecular analysis were carried out to unequivocally assign this strain to the *putida* species. Aranda-Olmedo *et al.* [14] showed that a conserved 35 bp repetitive extragenic palindromic (REP) sequence is specifically associated with *P. putida* strains. PCR analysis was performed using chromosomal DNA of HB3267 as a template and a primer based on the previously defined REP sequence [14]; and a positive amplicon was obtained. The same assay was performed with KT2440 as a positive control and *E. coli* as a negative control, with the expected results. The presence of the REP sequence in the genome of strain HB3267 suggested the original assignment of this strain to the species *P. putida* based on API classification was correct. To further confirm this result 16S rDNA amplification was carried out and the whole gene encoding the 16S sequence was obtained. Sequence comparison confirmed that HB3267 closest 16S genetic homologues all belong to the *P. putida* species. To establish a relationship with other *P. putida* strains, multi-locus sequence typing assays (MLST assays) were carried out using the housekeeping genes-*rpoD, gyrB, trpF, edd* and *recA*; these analyses confirmed that the gene products of HB3267 exhibited the highest identity with the gene products of several *P. putida* strains. Phylogenetically, strain HB3267 was closest to *P. putida* S16, a nicotine degrader isolated from a field under continuous tobacco cropping [23], [24] (Figure S1).

### Antibiotic resistance profile of P. putida strain HB3267

A distinctive characteristic of the HB3267 strain was its apparent multidrug resistance compared to other *P. putida* from the same hospital collection. Disk inhibition and MIC assays were performed with the most frequently used laboratory/clinical antibiotics to obtain quantitative data; for comparison we used *P. putida* KT2440R, a well characterized strain as a control (Table 1). Antibiogram assays showed that *P. putida* HB3267 was resistant to most of the 31 antibiotics tested in this study (Table 1). The exceptions were the aminoglycoside amikacin, as well as rifampicin and nitrofurantoin. A remarkable discovery was that HB3267 was resistant to all fluoroquinolones tested (ciprofloxacin, norfloxacin, pefloxacin and ofloxacin), while KT2440R showed high sensitivity to these antibiotics. The same was true for most aminoglycoside antibiotics tested (gentamycin, kanamycin, neomycin, streptomycin and netilmicin), with the exception of amikacin as mentioned.. The two strains also differed in their resistance to polymyxin B, colistin, cefotaxime, amoxicillin, imipenem, cephalosporin and ceftazidime, being that HB3267 was resistant to all of them while KT2440 was sensitive. MIC assays revealed that HB3267 was highly resistant to aminoglycoside antibiotics such as gentamycin, kanamycin and spectinomycin being, depending of the medium employed, at least 220-fold, 75-fold and 333-fold more resistant than KT2440R, respectively. The HB3267 strain was also much more resistant to polyketide antibiotics such as tetracycline (23.-fold more resistant), quinolone antibiotics such as nalidixic acid (67-fold), β-lactams such as ampicillin (30-fold), and bacteriostatic antibiotics such as chloramphenicol (3-fold). *Pseudomonas putida* KT2440R is a spontaneous mutant obtained by exposure to rifampicin [25], which explains why the MIC concentration for this antibiotic was 32-fold higher for KT2440R than for HB3267 (Table 2). Compared to other pseudomonad clinical isolates, the HB3267 strain was more resistant (in terms of range of antibiotic and MICs) than *P. aeruginosa* PAO1. For example, HB3267 was 5000 times more resistant to gentamicin than *P. aeruginosa* PAO1 [26].

**Table 1:** Antibiogram assay.

Antibiotic, concentration (mg)	Strain		Antibiotic group
	KT2440R*	HB3267*	
Ciprofloxacin, 5	2.8	0	fluoroquinolone
Norfloxacin, 10	2.5	0	fluoroquinolone
Pefloxacin, 5	1.9	0	fluoroquinolone
Ofloxacin, 5	1.8	0	fluoroquinolone
Nalidixic acid, 30	0	0	quinolone
Erithromycin, 15	0	0	macrolide
Gentamycin, 10	1.8	0	aminoglycoside c
Kanamycin, 30	2	0	aminoglycoside
Neomycin, 30	1.8	0	aminoglycoside
Streptomycin, 10	0.9	0	aminoglycoside
Amikacin, 30	0	1.7	aminoglycoside
Netilmicin, 30	1.4	0	aminoglycoside
Tetracycline, 30	0	0	polyketide
Polymyxin B, 300	1.3	0	polymyxin
Colistin, 50	1.5	0	polymyxin
Trimethoprim, 20	0	0	dihydrofolate reductase inhibitors
Chloramphenicol, 30	0	0	bacteriostatic
Amoxycillin, 25	1	0	ß-lactam (penicillin)
Carbenicillin, 100	0	0	ß-lactam (penicillin)
Ticarcillin, 70	0	0	ß-lactam (penicillin)
Piperacillin, 10	0	0	ß-lactam (penicillin)
Ampicillin, 10	0	0	ß-lactam (penicillin)
Imipemen, 10	2.8	0	ß-lactam (carbapenem)
Cefotaxime, 30	1.5	0	ß-lactam (cephalosporin)
Ceftazidime, 30	1.6	0	ß-lactam (cephalosporin)
Ceftriaxone, 30	0	0	ß-lactam (cephalosporin)
Sulfonamide G, 20	0	0	sulfonamides
Rifampicin, 30	0.6	1.6	rifamycin
Vancomycin, 30	0	0	glycopeptide
Esperamicin, 100	0	0	chromoprotein enediyne
Nitrofurantoin, 300	1.8	2.4	

Numbers indicate the size of the inhibition halo surrounding the antibiotic disc in cm.

Data are the average of 3 assays performed in duplicate with standard deviation below 5% of the given values.

**Table 2:** MIC and MBEC assays with different *Pseudomonas putida* strains.

	MIC (LB)		MIC (M9)		MIC (M-H)		MBEC	
Antibiotic	HB3267	KT2440R	HB3267	KT2440R	HB3267	KT2440R	HB3267	KT2440R
Tetracycline	200	8	450	30	350	15	>10000	2500
Kanamycin	3125	10	1500	10	1500	20	>25000	1600
Gentamicin	10000	20	5000	2	5500	25	>10000	1250
Nalidixic acid	2000	30	>1800	25	>1800	30	>10000	625
Spectinomycin	10000	30	>10000	10	>10000	15	>50000	1250
Rifampicin	62	2000	5	600	10	800	1250	5000
Choramphenicol	1000	376	1200	250	1000	300	>30000	1800
Ampicillin	10000	625	10000	600	10000	600	>100000	12500
Fluoroantimicin	1560	>3000	nt	nt	nt	nt	25000	nt
Amikacin	16	>100	1	2.5	1	2	50	625
Ceftriaxone	325	10	300	7.5	300	10	12500	1600
Norfloxacin	220	10	220	1	240	10	5000	75

Numbers indicate the MIC concentration (µg/ml) and MBEC concentrations (µg/ml) required to inhibit 90% growth and for biofilm eradication.

nt, not tested.

Horii *et al.* [27] analyzed the susceptibility of five clinical isolates of *P. putida* (from patients with acute, repetitive or chronic urinary tract infections) to fluoroquinolones. Similar to HB3267, four of the five isolates were resistant to fluoroquinolones, but in contrast with HB3267, all isolates were susceptible to aminoglycosides. MICs assays revealed that the resistance of HB3267 was at the same range than the described *P. putida* clinical isolates and at least 22-fold more than KT2440 [27]. Treviño *et al.* [28] also isolated two *P. putida* strains from immunocompromised patients. These two strains, as for the five isolates of Horii *et al.* [27], and the HB3267 strain we describe here, are susceptible to amikacin; these results indicate that this antibiotic could be used for treatment of multidrug-resistant *P. putida* strains. Resistance of *P. putida* clinical isolates to β-lactams [26], [27], [28], [29], aminoglycosides [26], [27], [30], and fluoroquinolones has been described [26], [27], [31], [32]; however, it should be noted that the minimal inhibitory concentrations found for HB3267 are much higher than what has been reported for other clinical isolates. HB3267 is the only *P. putida* clinical isolate reported to be resistant to tetracycline. The HB3267 strain is also the only *P. putida* clinical isolate described to be resistant to the biocide sulfonamide, trimethoprim/sulfamethoxazole and colistin, which is a characteristic often associated to *P. aeruginosa* [33], [34], [35].

It is known that *P. putida* strains are able to form biofilms on biotic and abiotic surfaces [25], [36]. Bacterial cells in biofilms are often more resistant to antibiotics than planktonic cells. We used the O'Toole and Kolter approach to produce biofilms of *P. putida* HB3267 which were flat and dense and similar to those produced by the KT2440 strain [36]. We then determined the Minimal Biofilm Eradication Concentration (MBEC) as described by Ceri *et al.* [19]. We found that both strains, KT2440 and HB3267 were much more resistant forming biofilms than living as planktonic cells, but most of the antibiotic tested, such as gentamicin, ampicillin, tetracycline, kanamycin, nalidixic acid, spectinomicyn and chloramphenicol, that were able to eradicate at high concentrations the KT2440 biofilms, were unable to eradicate those formed by HB3267. Antibiotics that were effective (rifampicin, fluoroantimicin, amikacin, ceftriaxone, and norfloxacin were required in 3- to 40-fold higher concentrations than those required to inhibit more than 90% growth of planktonic cells (Table 2).

### Gaining insight into antibiotic resistance through whole genome sequencing of HB3267

The total genome size of the *P. putida* HB3267 strain is 5908671 bp with a G+C content of 63.61%, which is similar to the published genomes of other *P. putida* strains. The HB3267 genome comprises a chromosome of 5829171 bp (Gene Bank CP003738) and a plasmid of 80360 bp named pPC9 (Gene Bank CP003739). The genome of HB3267 contains 5261 ORFs of which 5196 encode proteins. The other ORFs code for 61 tRNAs, 4 5S rRNAs, 4 16S rRNAs and 4 23S rRNAs. All essential conditional genes identified in KT2440 [4], [37] are present in the genome of HB3267, and functions were assigned to almost 75% of the total genes which encode proteins.

**Figure 1:**
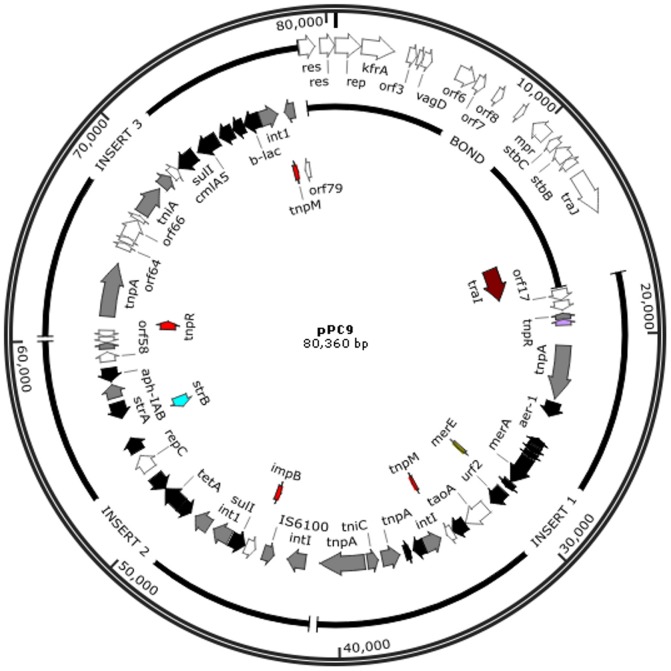
The pPC9 map. Genetic organization of pPC9, in white, genes forming the backbone of pPC9, in grey genes from the insert with homology to genes related to transposition, in black genes from the insert with antibiotic resistance function.

**Figure 2:**
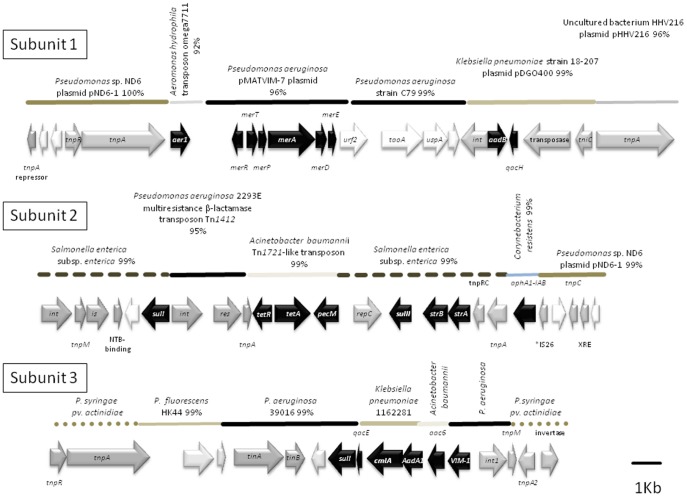
Genetic organization of the pPC9 “insert.” Black arrows represent genes with functions related to antibiotic resistance. In grey are genes with functions related to transposition and insertion machinery. Non-colored genes are those that encode hypothetical proteins, those with unknown function, or those with functions unrelated to antibiotic resistance, transposition or integration. Horizontal lines over genes represent DNA homology to different microorganisms, and percentages indicate the degree of homology.

Analysis of the sequence of the pPC9 plasmid revealed that this plasmid has a modular structure with 90 open reading frames (ORFs) that are distributed across several domains (Figure 1). The pPC9 backbone (38 kb) primarily contains genes for plasmid-related functions such as those required for replication and partitioning [38]. (Figure 1), and it exhibits high similarity to the backbone of pCT14 from *Pseudomonas* sp. CT14, a strain isolated from activated sewage sludge, while the “insert” region is made of several transposon-like elements bearing genes related to transposition and resistance to multiple antibiotics (Figure 2).

The backbone of pPC9 (Figure 1), bears only two putative conjugation genes (*traJ,I*), lacking most of the genes that would be necessary for self-mobilization. In agreement with this is that in biparental conjugation experiments this plasmid was unable to be transferred from HB3267 to other *P. putida* strains. Triparental mating experiments using the *Escherichia coli* pRK600 plasmid or the *P. putida* pWW0 plasmid as a helper also showed the inability of pPC9 to be transferred to *P. putida* strains. However, in experiments with pWW0, recombination events between pPC9 and pWW0 were detected; with transconjugants bearing a plasmid that conferred the ability to grow on toluene from pWW0, and streptomycin and tetracycline resistance from pPC9. This event occurred at a rate of 10^−4^ transconjugants per donor. This result confirms the presumption made by Yomoda *et al.* [11] that the existence of *P. putida* resident species in in-patients can facilitate the spread of drug resistance genes via horizontal gene transfer.

The “insert” region of the pPC9 plasmid is a mosaic of recruited DNA from different microorganisms. The antibiotic resistant determinants of pPC9 are grouped within three subregions as illustrated in Figure 2, surrounded by genes related to transposition and/or integration. A potential source of this DNA were closely related *Pseudomonas* strains such as the nonhuman pathogen *Pseudomonas* sp. ND6, isolated from industrial wastewater [39]; *Pseudomonas syringae* pv. *actinidiae*, which is the causal agent of bacterial canker of green-fleshed kiwifruit [40]; and *P. fluorescens* HK44, which colonizes plant roots and degrades phenolic compounds [41]. Within this “insert” region, DNA fragments could have also been acquired from human opportunistic pathogens or pathogens, such as *P. aeruginosa, Aeromonas hydrophila, Acinetobacter baumannii, Corynebacterium resistens* and Enterobacteriaceae (*Klebsiella pneumoniae*, *Salmonella enterica*) (Figure 2). This mosaic suggests that HB3267 may have been a resident of different environments, such as water, soil, plant rhizospheres and the human body, or that it has exchanged DNA with these potential microorganisms in different habitats.

### Antibiotic resistance determinants encoded only on the HB3267 chromosome

#### Quinolones and fluoroquinolones

Quinolones and fluoroquinolones are chemotherapeutic bactericidal drugs that eradicate bacteria by interfering with DNA replication. Quinolones enter into cells through porins and their targets are DNA gyrases and topoisomerases [42]. Point mutations in *gyrA* and *gyrB* (DNA gyrase subunits), and *parC* and *parE* (topoisomerase IV subunits) are associated with resistance to fluoroquinolone antibiotics [43]. In the HB3267 strain, GyrA showed a series of amino acid replacements with respect to the GyrA protein of fluoroquinolone-sensitive strains; HB3267 shows a Thr83Iso mutation in GyrA, which is known to lead to fluoroquinolone resistance in other clinic isolates of *P. putida* as HU2001-412 strain [27], that is present also in *P. aeruginosa* LESB58; only a unique difference in the case of HB3267 was a Val to Gly change at residue 894 (Figure S2A). In the case of GyrB, a polymorphism unique to HB3267 and the resistant HU2001-412 strain was Glu468Asp, also known to lead fluoroguinolone resistance [27] (Figure S2B). For ParC, the substitution Ser87Leu was present in HB3267 and *P. aeruginosa* PA7; however, for the other topoisomerase IV subunit ParE no substitution was found. The unique polymorphism in GyrA and/or GyrB and/or ParC may be responsible for the resistance of HB3267 against these antibiotics, but this hypothesis requires further testing using site specific mutants and gene complementation experiments.

#### Cationic antimicrobial peptides

Strain HB3267 is more resistant to polymyxin B than KT2440R (Tables 1 and 2) and other strains [44]. LPS is one of the surface elements that influence resistance to cationic peptides in P. aeruginosa [45]. Vaara (1993) described that the firA gene product, involved in lipid A biosynthesis was relevant in the resistance of E. coli and S. typhimurium to polymyxin B [46]. Strain HB3267 harbours two copies of this gene which encodes for a UDP-3-O-3-hydroxymyristoyl glucosamine N-acyltransferase that is 70% identical to the FirA of Klebsiella pneumoniae. The higher copy number of firA may provide an explanation for the HB3267 strain's higher resistance to polymyxin B than in other strains of P. putida.

*Antibiotic resistance determinants encoded on the chromosome of HB3267 and its resident pPC9 plasmid.*

#### Aminoglycosides

Aminoglycosides bind to the A-site of the 30S subunit of bacterial ribosomes disturbing elongation of the peptide chain. Wei et al. (2011) demonstrated using data obtained from phenomics, transcriptomics and proteomic analysis that resistance to aminoglycosides in the P. aeruginosa PAO1 strain is multifactorial including the presence of mutations in chromosomal genes such as the phoP and phoQ, as well as in the mexZ gene encoding a repressor of the mexXY genes encoding an efflux pump [47]. When all the sequences of phoQ from aminoglycoside sensitive Pseudomonas strains were compared to the resistant HB3267 strain, the Gly365Arg polymorphism was exclusive to HB3267(Figure S2C). In the case of PhoP, Ser21Gly (exception P. putida S16 and P. entomophila L48), and Arg204His (exception P. putida S16) changes were found but none were exclusive of HB3267. No clear homolog to mexZ was present in the chromosome of HB3267.

HB3267 sensitivity and KT2440 resistance to amikacin may be explained by multifactorial differences in expression of chromosomal genes involved in cell permeability, LPS synthesis, efflux pumps and chemical modification [48]. Vaziri *et al.* (2011) described the existence of aminoglycoside modifying enzymes that are encoded by plasmids as the primary resistance mechanism employed by *P. aeruginosa* against these antibiotics [49]. The pPC9 plasmid carries genes that encode 6 aminoglycoside modifying enzymes that are not present in the genome of *P putida* strains sensitive to aminoglycosides. Therefore, these aminoglycosidases likely contribute to the resistance phenotype of HB3267. One of these aminoglycosidases was 100% identical to the *aadB* gene product of *P. aeruginosa* (Figure 2 subunit 1). This protein has 2″-aminoglycoside nucleotidyltransferase activity, and has been proposed to be responsible for bacterial resistance to the aminoglycosides dibekacin, gentamicin, kanamycin, sisomicin and tobramycin [50]. Another protein was 100% identical to StrA from *Salmonella enterica* that has aminoglycoside 3′-phosphotransferase activity and a third protein was 100% identical to StrB from *Acinetobacter baumannii*, which has aminoglycoside 6′-phosphotransferase activity (Figure 2 subunit 2). Both of these proteins have been traditionally associated with resistance to the aminoglycoside streptomycin [51]. We also found a gene that encodes for a protein that is 100% identical to AphA1-IAB (Figure 2 subunit 2) from *Corynebacterium striatum*, which is an aminoglycoside 3′-phosphotransferase involved in the inactivation of aminoglycoside antibiotics such as kanamycin, neomycin, neamine, and ribostamycin [52]. Another gene coding for a protein with 99% identity to the *aadA1* gene product from *Escherichia coli* (Figure 2 subunit 3) is also present. AadA1 is an aminoglycoside-3′-adenylyltransferase that confers resistance to streptomycin and spectinomycin [53]. Finally, there is also a gene that codes a protein which is 98% identical to Aac6 from *Enterobacter cloacae*, an aminoglycoside phosphotransferase that confers resistance to netilmicin and tobramycin [54]. It therefore appears that the pPC9 plasmid has recruited a number of aminoglycoside modifying enzymes from different origins.

#### Tetracyclines

**Figure 3:**
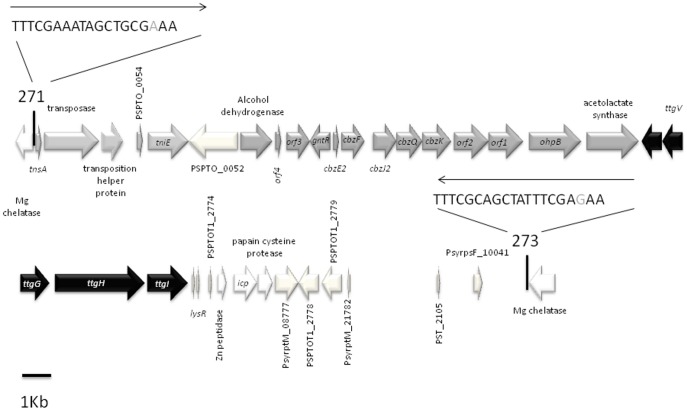
Location of the antibiotic and solvent efflux *ttgGHI* pumps within a genomic island in the chromosome of strain HB3267. *ttgGHI* efflux genes are indicated in black; genes involved in transposition events are in light grey; the *cbz* operon, which is involved in chlorobenzene degradation, is in medium grey. Vertical lines indicate the insertion point; arrows above the sequence indicate the inverted repeat sequences of the *Tn552*-like transposon, which are within the magnesium chelatase.

Tetracyclines bind to the 30S subunit of microbial ribosomes. They inhibit protein synthesis by blocking the attachment of charged aminoacyl-tRNAs to the A site on the ribosome. Thus, they prevent introduction of new amino acids to the nascent peptide chain [55]. Several mechanisms have been described by which bacteria gain resistance to tetracycline, namely, extrusion of tetracycline via efflux pumps, changes in ribosome proteins so that tetracycline no longer binds, and chemical inactivation of tetracyclines. Tetracycline efflux is the most efficient mechanism of resistance to this antibiotic for Gram-negative bacteria [56]. The resistance of P. putida KT2440 to tetracycline is linked to the RND TtgABC efflux pump [57]. The TtgABC pump is also responsible for resistance to a broad range of antibiotics such as β-lactams, nalidixic acid, and chloramphenicol [57], [58]. The genes which encode the TtgABC pump are located on the chromosome of HB3267. A secondary role in tetracycline resistance was assigned to the TtgGHI efflux pump, which is located on the pGRT1 plasmid in P. putida DOT-T1E [59], although the primary role of this pump in P. putida DOT-T1E appears to be solvent extrusion [58], [59], [60]. The ttgGHI genes are present in the chromosome of HB3267 and the operon is located in a 44 Kb genomic island (Figure 3) with no homology to the chromosomal sequences of other P. putida strains or with the rest of pGRT1 plasmid [60]. The total G+C content of this island is 55%, a value lower than that of the rest of chromosome. The gene that encodes the repressor of this ttgGHI operon, the TtgV protein is 89% identical to that of DOT-T1E.

It should be noted that the pPC9 plasmid also encodes another tetracycline efflux pump of the TetA type, which is 100% identical to the *Acinetobacter baumannii Tn1721*-like transposon (Figure 2 subunit 2) [61]. This gene is not present in the genome of KT2440R; the presence of multiple tetracycline efflux pumps supports the data which shows that HB3267 exhibits higher levels of resistance to tetracycline than other strains.

#### β-lactams

RND efflux pumps and β-lactamases are key players in the resistance of Pseudomonads to β-lactam antibiotics. The HB3267 chromosome carries 10 β-lactamase genes that are also present in KT2440, explaining the resistance of both strains to penicillin-derived antibiotics such as ampicillin, carbenicillin, ticarcillin and piperacillin, and the cephalosporin ceftriaxone. Livermore suggested that the phenotype of resistance to the cephalosporin cefotaxime and ceftazidime of some Pseudomonas clinical isolates was mediated by the action of the chromosomal ampC gene that codes for a β-lactamase [62]. This gene is present in KT2440, which is sensitive to these antibiotics. Two single nucleotide polymorphisms were found in the ampC gene of HB3267 when compared to the sensitive KT2440 strain, namely Pro148Ala and Gly263Arg, which may explain the HB3267 resistance to these antibiotics.

We found that the pPC9 plasmid bears 2 additional β-lactamase genes, one that codes for an enzyme that is 95% identical to AER-1 from *Aeromonas hydrophila* (Figure 2 subunit 1)—a protein that can efficiently hydrolyze carbenicillin [63]; as well as a gene that codes for a protein that is 100% identical to VIM-1 from *Klebsiella pneumonia* (Figure 2 subunit 3), which is involved in the carbapenem-resistant phenotype of that microorganism [64].

#### Chloramphenicol

**Figure 4:**
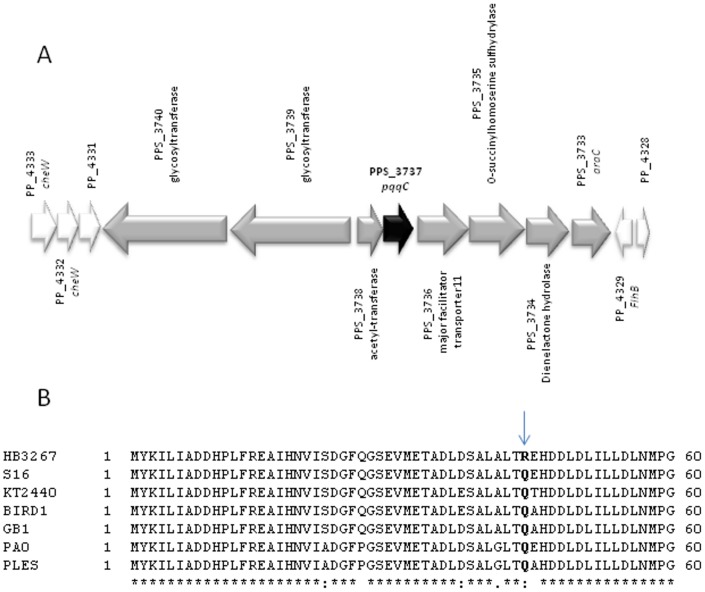
Potential chromosomal determinants for chloramphenicol resistance of HB3267. (A) In black, chromosomal location of the additional *pqqC* gene of HB3267.; in grey, the region of the HB3267 chromosome that is not present in KT2440; in white, genes in synteny with KT2440. (B) Protein alignment of AgmR from *P. putida* HB3267 (**HB3267**, Locus B479_11475), *P. putida* S16 (**S16**, PPS_2213), *P. putida* KT2440 (PPS_2213, PP_2665), *P. putida* BIRD-1 (**BIRD1**, PPUBIRD1_3011), *P. putida* GB-1 (**GB1**, PputGB1_3138), *P. aeruginosa* PA7 (PA7, PSPA7_3317), and *P. aeruginosa* PAO1 (**PAO1**, PA1978) strains. Amino acid mutations referred to in the text are indicated in bold.

Chloramphenicol is a bacteriostatic antimicrobial that functions by inhibiting bacterial protein synthesis. P. putida KT2440 is a chloramphenicol-resistant bacterium that is able to grow in the presence of this antibiotic at a concentration of up to 25 µg/ml. Genomic analysis revealed that the TtgABC efflux pump and biosynthesis of pyrroloquinoline (PQQ) were involved in chloramphenicol resistance [65]. These genes are present in the chromosome of the HB3267 strain, which also shows high resistance to this antibiotic. An additional pqqC (coenzyme PQQ synthesis protein C) gene is present in the chromosome of the HB3267 strain, which is also present in the close relatives P. putida S16 and Pseudomonas sp. TJI-51, but not in KT2440 (Figure 4A). The AgmR regulator (PP2665) controls the expression of the pqq genes and the operon encoding the ABC extrusion pump [65]. Up to three polymorphisms were present in PqqC of HB3267 when compared to other P. putida strains, namely HisGln44 (Figure 4B), His142Leu and Ala116Gly. Per se, these mutations and the presence of an additional copy of the pqqC gene could explain in part the high resistance of HB3267 to chloramphenicol. In addition plasmid pPC9 encodes a protein that is 99% identical to CmlA from Aeromonas caviae (Figure 1 subunit 3), an efflux pump that expels chloramphenicol and ethidium from the cells [66].

### Antibiotic resistance determinants encoded only on the pPC9 plasmid –

#### Sulfonamides

Sulfonamides were the first compounds used as chemotherapy drugs and have been used as antibacterial agents since the 1930's. In bacteria, sulfonamides act as competitive inhibitors of the dihydropteroate synthetase (DHPS), an enzyme involved in folate synthesis. As such, the compounds cause microorganisms to become “starved” of folate and die [67]. Plasmid-mediated sulfonamide resistance in Gram-negative bacteria is very frequently found in clinical isolates and often in combination with other antibiotic resistance traits. The plasmids generally express alternative dihydropteroate synthases, the Sul proteins, which confer resistance to the drug [68]. In pPC9, three genes homologous to those encoding Sul proteins were found: one with 97% identity to the SulI protein of P. aeruginosa (Figure 2. subunit 2), another with a 100% identity with SulII of diverse enteric bacteria (Figure 2. subunit 2), and finally a gene coding a protein with 99% identity to SulI of E. coli (Figure 2. subunit 3).

Our results show that strain *P. putida* HB3267, isolated from a deceased in-patient in a French hospital, is resistant to the majority of antibiotics and biocides used in laboratories and hospitals (aminoglycosides, ß-lactam antibiotics, cationic peptides dihydrofolate reductase inhibitors, fluoroquinolones, quinolones, glycopeptide antibiotics, macrolides, polyketide, and sulfonamides). This broad range of resistance is rarely found in clinical isolates. Another relevant finding is that MICs for these antibiotics in planktonic cells were much higher for HB3267 than that of multidrug-resistant strains of *Pseudomonas aeruginosa*. Sequencing of the genome of HB3267 revealed that the determinants of multiple resistances are located chromosomally and on the plasmid pPC9. Regions of the plasmid bearing multidrug resistant genes show high homology with DNA from environmental microorganisms as well as from human opportunistic and true human pathogens indicating both contact and DNA exchange between the HB3267 strain and these environmental and clinically relevant microorganisms. The pPC9 plasmid carries integrons and transposons where the antibiotic resistant determinants are grouped. We have shown that pPC9 is not self-transmissible but transfer of the antibiotic resistant genes from pPC9 to other microbes can be mediated by shuttle vectors, such as the TOL plasmid pWW0. The results presented in this work support the notion that the acquisition of new antibiotic and biocide resistant traits by opportunistic human pathogens, may arise from the cohabitation in the human body of pathogens with new multidrug-resistant “residents”, such as the HB3267 strain.

## Supporting Information

Figure S1 **Phylogenetic tree comparing** ***gyrB*** **genes of** ***Pseudomonas strains*** **.** Phylogram constructed using the platform Phylogeny.fr. which is a combination of a predefined pipeline using leading programs that include MUSCLE, Gblocks, PhyML and TreeDyn [23]. *P. aeruginosa* PAO-1 (NC_018080), *P. fluorescens* F113 (NC_016830), *P. monteilii* BCRC 17520 (FJ418641), *P. putida* BIRD-1 (NC_017530), *P putida* GB-1 (NC_010322), *P. putida* KT2440 (NC_002947), *P. putida* HB3267 (CP003738), *P. putida* S16 (NC_015733).
(TIF)

Figure S2 **Protein alignment of GyrA (A), GyrB (B) and PhoQ (C) from** ***P. putida*** **HB3267 (HB3267, Locus B479_00265, B479_06830, B479_20445, respectively),** ***P. putida*** **S16 (S16, PPS_1408, PPS_0012, PPS_4028),** ***P. putida*** **KT2440 (KT2440, PP_1767, PP_0013, PP_1187),** ***P. putida*** **BIRD-1 (BIRD1, PPUBIRD1_3846…., PPUBIRD1_1228),** ***P. putida*** **GB-1 (GB1, PputGB1_1358, PputGB1_0006, PputGB1_4229) strains and** ***P. aeruginosa*** **LESB58 (LESB PLES_19001, PLES_00031, PLES_41411),** ***P. aeruginosa*** **PAO1 (PAO1, PA3168, PA0004, PA1180) strains.** Amino acid changes referred to in the text are indicated in bold; “*”Identical residues, “:” conservative substitutions and “.” semiconservative substitutions.
(TIF)

Table S1 **RND efflux pumps in P. putida KT2440 and their orthologs in the HB3267 strains.**
(TIF)
