# Supplementary figures and images for: Antibiotic Resistance Determinants in a Pseudomonas putida Strain Isolated from a Hospital
Source: PLoS One. 2014 Jan 17;9(1):e81604. doi: 10.1371/journal.pone.0081604 (PMC3894933; doi:10.1371/journal.pone.0081604)

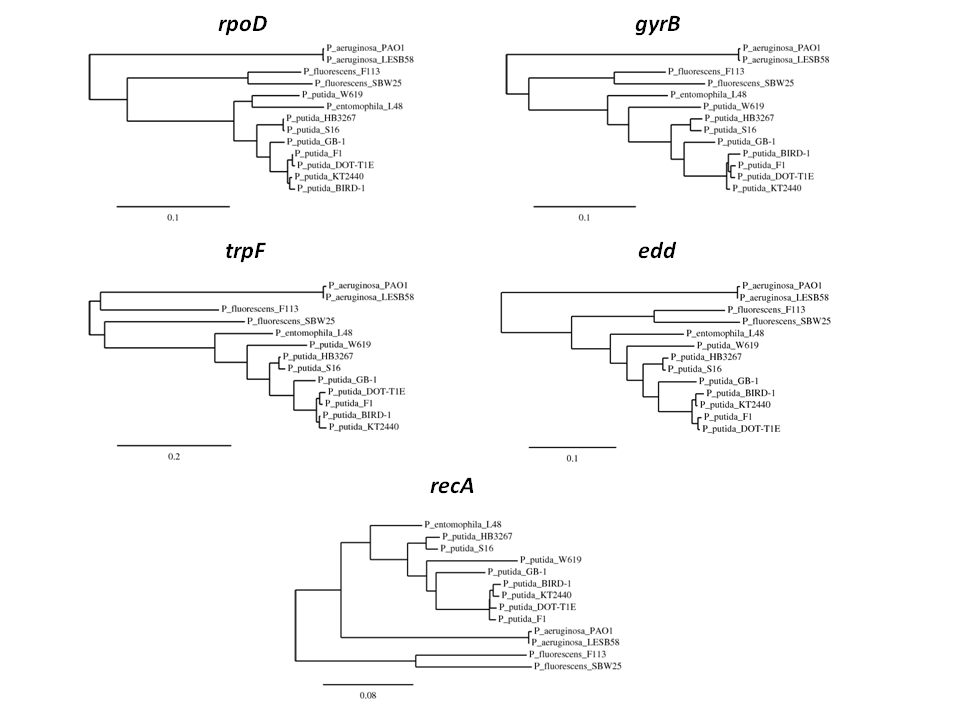

Supplement: Figure S1 — Phylogenetic tree comparing gyrB genes of Pseudomonas strains . Phylogram constructed using the platform Phylogeny.fr. which is a combination of a predefined pipeline using leading programs that include MUSCLE, Gblocks, PhyML and TreeDyn [23]. P. aeruginosa PAO-1 (NC_018080), P. fluorescens F113 (NC_016830), P. monteilii BCRC 17520 (FJ418641), P. putida BIRD-1 (NC_017530), P putida GB-1 (NC_010322), P. putida KT2440 (NC_002947), P. putida HB3267 (CP003738), P. putida S16 (NC_015733). (TIF) [file pone.0081604.s001.tif]

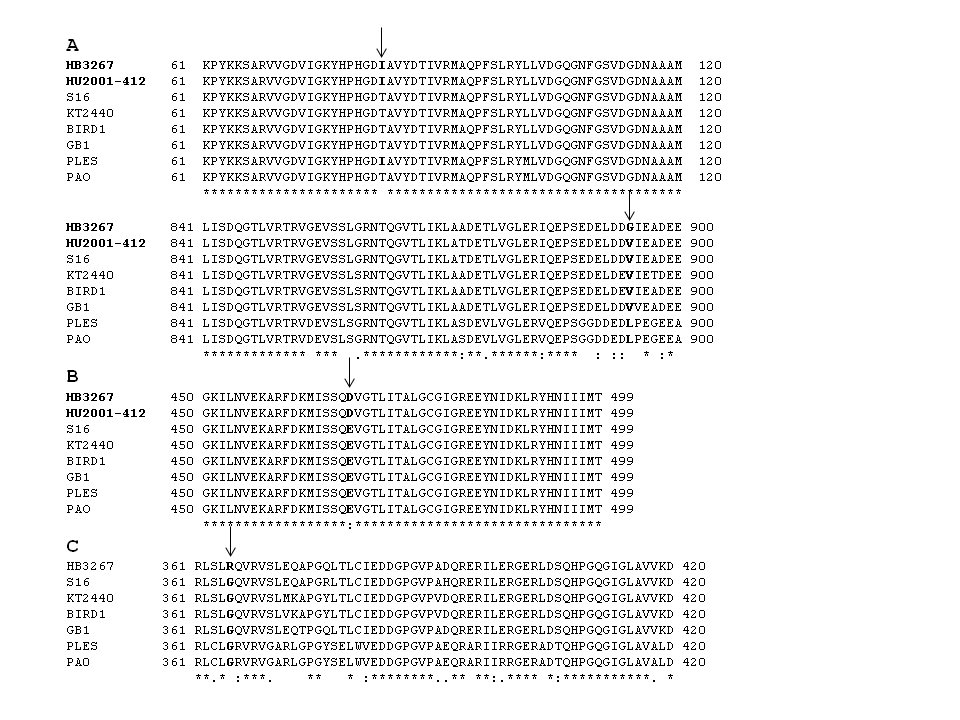

Supplement: Figure S2 — Protein alignment of GyrA (A), GyrB (B) and PhoQ (C) from P. putida HB3267 (HB3267, Locus B479_00265, B479_06830, B479_20445, respectively), P. putida S16 (S16, PPS_1408, PPS_0012, PPS_4028), P. putida KT2440 (KT2440, PP_1767, PP_0013, PP_1187), P. putida BIRD-1 (BIRD1, PPUBIRD1_3846…., PPUBIRD1_1228), P. putida GB-1 (GB1, PputGB1_1358, PputGB1_0006, PputGB1_4229) strains and P. aeruginosa LESB58 (LESB PLES_19001, PLES_00031, PLES_41411), P. aeruginosa PAO1 (PAO1, PA3168, PA0004, PA1180) strains. Amino acid changes referred to in the text are indicated in bold; “*”Identical residues, “:” conservative substitutions and “.” semiconservative substitutions. (TIF) [file pone.0081604.s002.tif]

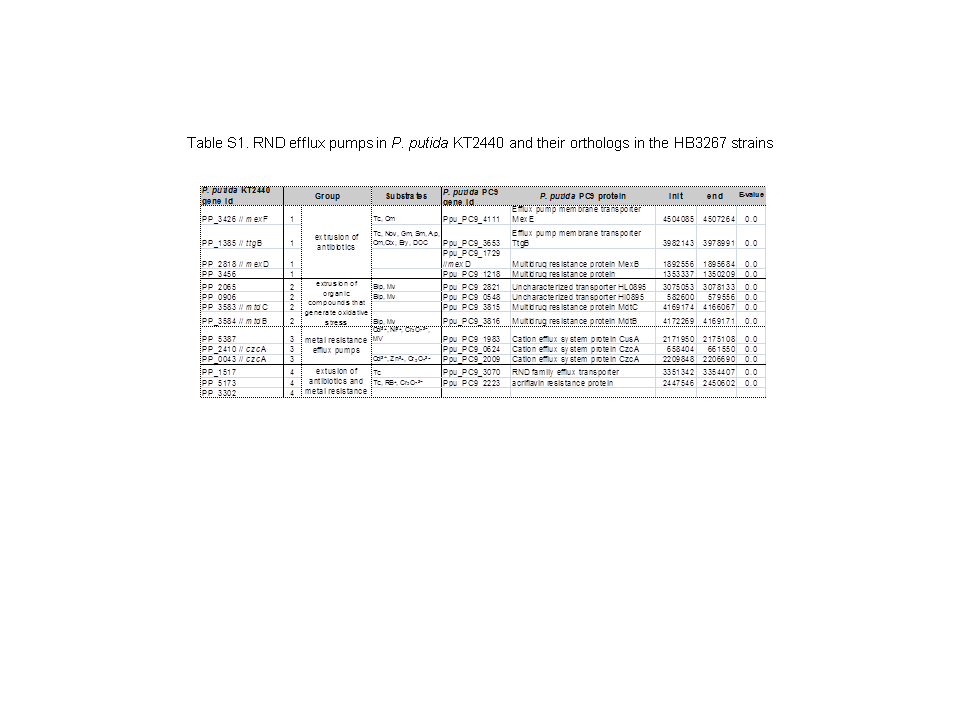

Supplement: Table S1 — RND efflux pumps in P. putida KT2440 and their orthologs in the HB3267 strains. (TIF) [file pone.0081604.s003.tif]
